# Antifibrotic effect of lung-resident progenitor cells with high aldehyde dehydrogenase activity

**DOI:** 10.1186/s13287-021-02549-6

**Published:** 2021-08-23

**Authors:** Hiroshi Takahashi, Taku Nakashima, Takeshi Masuda, Masashi Namba, Shinjiro Sakamoto, Kakuhiro Yamaguchi, Yasushi Horimasu, Shintaro Miyamoto, Hiroshi Iwamoto, Kazunori Fujitaka, Hironobu Hamada, Noboru Hattori

**Affiliations:** 1 Department of Molecular and Internal Medicine, Graduate School of Biomedical and Health Sciences, Hiroshima University, 1-2-3 Kasumi, Minami-ku, Hiroshima, 734-8551 Japan; 2 Department of Physical Analysis and Therapeutic Sciences, Graduate School of Biomedical and Health Sciences, Hiroshima University, 1-2-3 Kasumi, Minami-ku, Hiroshima, 734-8553 Japan

**Keywords:** Aldehyde dehydrogenase, Bleomycin, Cell therapy, Profibrotic cytokines, Pulmonary fibrosis, Stem cells

## Abstract

**Background:**

Aldehyde dehydrogenase (ALDH) is highly expressed in stem/progenitor cells in various tissues, and cell populations with high ALDH activity (ALDH^br^) are associated with tissue repair. However, little is known about lung-resident ALDH^br^. This study was performed to clarify the characteristics of lung-resident ALDH^br^ cells and to evaluate their possible use as a tool for cell therapy using a mouse model of bleomycin-induced pulmonary fibrosis.

**Methods:**

The characteristics of lung-resident/nonhematopoietic (CD45^−^) ALDH^br^ cells were assessed in control C57BL/6 mice. The kinetics and the potential usage of CD45^−^/ALDH^br^ for cell therapy were investigated in bleomycin-induced pulmonary fibrosis. Localization of transferred CD45^−^/ALDH^br^ cells was determined using mCherry-expressing mice as donors. The effects of aging on ALDH expression were also assessed using aged mice.

**Results:**

Lung CD45^−^/ALDH^br^ showed higher proliferative and colony-forming potential than cell populations with low ALDH activity. The CD45^−^/ALDH^br^ cell population, and especially its CD45^−^/ALDH^br^/PDGFRα^+^ subpopulation, was significantly reduced in the lung during bleomycin-induced pulmonary fibrosis. Furthermore, mRNA expression of ALDH isoforms was significantly reduced in the fibrotic lung. When transferred in vivo into bleomycin-pretreated mice, CD45^−^/ALDH^br^ cells reached the site of injury, ameliorated pulmonary fibrosis, recovered the reduced expression of ALDH mRNA, and prolonged survival, which was associated with the upregulation of the retinol-metabolizing pathway and the suppression of profibrotic cytokines. The reduction in CD45^−^/ALDH^br^/PDGFRα^+^ population was more remarkable in aged mice than in young mice.

**Conclusions:**

Our results strongly suggest that the lung expression of ALDH and lung-resident CD45^−^/ALDH^br^ cells are involved in pulmonary fibrosis. The current study signified the possibility that CD45^−^/ALDH^br^ cells could find application as novel and useful cell therapy tools in pulmonary fibrosis treatment.

**Supplementary Information:**

The online version contains supplementary material available at 10.1186/s13287-021-02549-6.

## Background

Tissue-resident stem cells are valuable in cell therapy and have been successfully used for immunomodulation, tissue regeneration, and tissue repair. Several trials using stem cell therapy have been performed to treat refractory diseases, with mesenchymal stem cells (MSCs) being the most frequently used cell type [1]. In particular, MSCs, shown to exhibit pluripotency toward the nonhematopoietic cell lineage, can be isolated from various organs, including the bone marrow, adipose tissue, skeletal muscle, and the umbilical cord [1]. Bone marrow-derived MSCs, isolated from the most orthodox cell source of MSCs [2, 3], have been shown to have immunomodulatory effects such as the inhibition of the proliferation of T-cells through secretion of anti-inflammatory cytokines and growth factors [4]. In a mouse model of bleomycin (BLM)-induced lung injury, administration of bone marrow-derived MSCs was reported to improve lung injury by exerting an anti-inflammatory effect [5]. With respect to lung resident stem cells, the Sca1^+^/CD45^−^/CD31^−^ cell population has been identified as lung tissue stem cells capable of differentiating into endothelial and lung epithelial cells in vitro. Moreover, when transferred into an elastase-induced lung injury mouse model, this population was demonstrated to significantly improve the survival rate and reverse lung damage [6]. Lung Hoechst 33342^dim^ side population (SP) cells are adult stem cells, which have also been identified to exhibit mesenchymal and epithelial potential [7]. Among the SP cells, the CD45^−^/CD31^−^ fraction has been reported to have the characteristics of lung resident MSCs, due to their ability to differentiate into smooth muscle, bone, fat, and cartilage [8, 9]. Furthermore, the number of lung resident SP cells was shown to be significantly reduced in mice with BLM-induced lung injury, and this reduction was correlated with the pathology of the lung injury. When administered intravenously into the lung, lung SP cell therapy was shown to reduce BLM-induced pulmonary fibrosis and pulmonary arterial hypertension [10]. These results suggest the existence of tissue-specific MSCs in the lung and their involvement in lung injury.

Aldehyde dehydrogenases (ALDH) are a group of enzymes that catalyze the oxidation of aldehydes to carboxylic acids, with 19 different isoforms in humans [11]. A cell population with high ALDH activity, called ALDH bright cells (ALDH^br^), is associated with the stemness of various normal tissues and is involved in tissue repair [12]. Moreover, ALDH^br^ isolated from the human bone marrow, reported to have a higher colony-forming capacity when compared to a cell population with low ALDH activity (ALDH^dim^) [13], was shown to be a progenitor population for epithelial, endothelial, and mesenchymal lineages [14]. When administered in a mouse model of myocardial infarction, ALDH^br^ collected from the human umbilical cord blood was demonstrated to enhance angiogenesis in the ischemic heart [15]. Given these findings, the existence of lung resident ALDH^br^ and its contribution to tissue repair were speculated; however, little is known about lung resident ALDH^br^. The objectives of this study were to clarify the characteristics of lung-resident ALDH^br^ and to evaluate its possible use as a tool for cell therapy in a mouse model of BLM-induced pulmonary fibrosis.

## Methods

### Animals and BLM-induced pulmonary fibrosis

This study, aimed at elucidating the characteristics of lung-resident ALDH^br^ and exploring its usage in cell therapy, was performed in accordance with the protocols approved by the Animal Ethics Committee of Hiroshima University (A19-122 and 28-29-2). In this study, pulmonary fibrosis was induced as previously described [16] in C57BL/6J mice (6–8-week-old young female mice and 52 week old aged female mice) which were purchased from Charles River Laboratories Japan (Yokohama, Japan). The mice were maintained in a specific pathogen-free environment and randomly assigned to BLM or control groups. In experiments performed to confirm the localization of transferred cells, C57BL/6-Gt (ROSA)26Sor < tm1.1 (H2B-mcherry) Osb > heterozygotic mice (mCherry mouse, BRC No. RBRC06036, RIKEN, Tokyo, Japan) [17] systemically expressing the mCherry protein in their nuclei were used as a donor population. On day 0, after intraperitoneal injection of mixed anesthesia with medetomidine hydrochloride (0.3 mg/kg body weight; Kyoritsu Seiyaku, Tokyo, Japan), midazolam (4 mg/kg body weight, Sandoz K.K., Tokyo, Japan), and butorphanol tartrate (5 mg/kg body weight, Meiji Seika Pharma, Tokyo, Japan), pulmonary fibrosis was induced by endotracheal injection of BLM (2 mg/kg of body weight, Nippon Kayaku, Tokyo, Japan). Control mice received the same amount (2 mL/kg body weight) of phosphate-buffered saline (PBS, Nacalai Tesque, Kyoto, Japan) alone. For survival analysis, a higher dose of BLM (5 mg/kg) was used. At 7 and 14 days after BLM administration, both lungs were removed from each animal and the lung tissue was assessed for hydroxyproline, and mRNA expression and subjected to flow cytometry and histological analysis.

### Cell isolation

The lungs were removed and minced in 1-mL Roswell Park Memorial Institute 1640 medium (Thermo Fisher Scientific, Waltham, MA, USA) supplemented with collagenase A (1 mg/mL, Roche, Basel, Switzerland), and incubated at 37 °C for 30 min. Following lysis of red blood cells with ACK Lysing Buffer (Life Technologies, Grand Island, NY, USA), the cells were resuspended in 2 mL of PBS containing 0.5% bovine serum albumin (Sigma-Aldrich, St. Louis, MO, USA) and 2 mM ethylenediaminetetraacetic acid (Sigma-Aldrich), and cell counting was performed.

### Antibodies and ALDH staining

Antibodies (all purchased from BioLegend, San Diego, CA, USA) used for flow cytometry and cell sorting are shown in Additional file 1. After staining for cell surface proteins using the aforementioned antibodies, ALDH activity was expressed as fluorescent intensity using the ALDEFLUOR™ Kit (STEMCELL Technologies Inc., Vancouver, Canada) according to the manufacturer’s protocol, as previously reported [18]. A separate tube containing 5 µL of diethylaminobenzaldehyde (DEAB, provided in the ALDEFLUOR™ Kit), a specific inhibitor of ALDH, was prepared to determine ALDH^br^ gating.

### Hoechst staining

Hoechst 33,342 staining of lung cells was performed as previously reported [19]. After suspended in 1 mL DMEM (Thermo Fisher Scientific) with 5% FBS (Sigma-Aldrich), 1.0 × 10^6^ cells were stained with 4 µL Hoechst 33,342 (Invitrogen, Carlsbad, CA, USA) alone or in combination with 30 µL verapamil (Sigma-Aldrich) for 90 min at 37 °C with mixing every 20 min during staining. Antibody and ALDH staining were performed as described above after Hoechst 33,342 staining.

### Flow cytometry and cell sorting

Flow cytometric analysis of lung cells was performed using the following method, referring to a previous report [20]. Flow cytometry and cell sorting were performed using the FACS Aria II system (BD Biosciences, San Jose, CA, USA) and LSRFortessa X-20 (BD Biosciences). Data were analyzed using the FACS Diva (BD Biosciences) and the FlowJo (version 10.7.1, BD Biosciences) software. For the isolation of ALDH^br^, unnecessary cell populations were pre-depleted using magnetic cell sorting (MACS) cell separation using a Stem Cell Pre-Enrichment kit (Miltenyi Biotec, Bergisch Gladbach, Germany) prior to FACS according to the manufacturer’s protocol. Cell sorting from mCherry-expressing donor mice and analysis of injected donor mCherry^+^ cells was performed using the SORP Aria (BD Biosciences) and LSRFortessa X-20 (BD Biosciences) systems, respectively.

### Cell culture and colony-forming assay

Sorted cells were seeded into 96-well plates at a density of 5–10 × 10^3^ cells/well and cultured in Dulbecco’s modified Eagle medium (DMEM, Thermo Fisher Scientific) and 10% fetal bovine serum (FBS, Sigma-Aldrich) supplemented with or without 20 ng/mL epidermal growth factor (EGF, BioLegend) or 20 ng/mL fibroblast growth factor-2 (FGF2, BioLegend) or both. The medium was changed every 3–4 days. For colony formation, 5.0 × 10^3^ cells were seeded into 6-well plates using MethoCult (STEMCELL Technologies Inc.). Consecutively, 2 to 3 weeks after the start of culture, the number of proliferated colonies was counted.

### Cell viability assay

Cells were seeded into 96-well plates at a density of 5.0 × 10^3^ cells/well and the medium was changed every 3–4 days. After 3–4 weeks from the start of the culture, cell proliferation was evaluated using Cell Counting Kit-8 (Dojindo, Kumamoto, Japan).

### Cell transfer to recipient mouse

Sorted 1.0 × 10^5^ CD45^−^/ALDH^br^ and CD45^−^/ALDH^dim^ cells were dissolved in 100 µL PBS and administered intravenously via the tail vein to recipient BLM-pretreated mice on day 2 (2 days after treatment with BLM). To confirm the localization of transferred cells, 5.0 × 10^4^ mCherry^+^ CD45^−^/ALDH^br^ and CD45^−^/ALDH^dim^ cells were administered intravenously into recipient BLM-pretreated C57BL/6 mice on day 2. On the following day and 5 days after the injection (on days 3 and 7), the recipient mice were sacrificed, and lung samples were subjected to flow cytometry and histology analyses.

### Hydroxyproline assay

The left lungs were removed and the sample was homogenized in 1 mL of PBS and hydrolyzed with 1 mL of HCl for 16 h at 120 °C. The supernatant was centrifuged at 10,000*g* for 5 min (Model 3740, KUBOTA, Tokyo, Japan), and 5 µL of the supernatant was aliquoted into a 96-well plate. After dispensing 5 µL hydroxyproline standard (Sigma-Aldrich) into each well of the 96-well plate, 5 µL citrate/acetate buffer (deionized distilled water supplemented with 238 mM Citric acid, Sigma-Aldrich, 1.2% glacial acetic acid, Sigma-Aldrich, 532 mM sodium acetate, Sigma-Aldrich, and 850 mM sodium hydroxide, Nacalai Tesque) and 100 µL chloramine T solution (1.0 mL deionized distilled water supplemented with 0.141 g chloramine T, Sigma-Aldrich, 1.0 mL 1-propanol, Sigma-Aldrich, and 8.0 mL citrate/acetate buffer) were added. After 30 min of incubation at 25 °C, 100 µL of Ehrlich's reagent (2.5 g 4-dimethylaminobenzaldehyde, Sigma-Aldrich, 9.3 mL 1-propanol, and 3.9 mL 70% perchloric acid, Sigma-Aldrich) was added and the mixture was incubated at 65 °C for 30 min. After 5 min at 25 °C, the absorbance was measured at 550 nm using a plate reader (iMARK, Bio-Rad, Hercules, CA, USA), as previously described [21].

### PCR and agarose gel electrophoresis

The sorted cells and the excised lungs were homogenized using 1 mL TRIzol reagent (Life Technologies) and total RNA was extracted using the RNeasy Mini Kit (QIAGEN, Venlo, Netherlands). The extracted RNA was reverse transcribed into cDNA using the High Capacity RNA-to-cDNA Kit (Applied Biosystems, Foster City, CA, USA). Real-time quantitative PCR was performed using the Applied Biosystems 7500 Fast Real-Time PCR System (Applied Biosystems) and the TaqMan Gene Expression Assays (Applied Biosystems) as previously described [16]. The expression of *Actb* (β-actin, Mm02619580_g1; Applied Biosystems) was used as an endogenous control. The TaqMan Gene Expression Assays were used as shown in Additional file 2. To distinguish the mCherry-heterozygotic mice the from wild-type mice, mouse-tail DNA was extracted using the DNeasy Blood and Tissue Kit (QIAGEN). The extracted DNA was subjected to PCR using the primers shown in Additional file 2. PCR conditions were as follows: 120 s at 94 °C, 10 s at 98 °C, 30 s at 60 °C, 120 s at 68 °C, repeated for 30 cycles. Amplified products were stained with SAFELOOK™ (Fujifilm Wako Junyaku, Osaka, Japan), and bands were confirmed using electrophoresis on a 1% agarose gel.

### Histological analysis

Lung tissue sections were fixed in 2% formalin solution (Nacalai Tesque, Kyoto, Japan) and embedded in paraffin, followed by hematoxylin–eosin (HE) and Masson’s trichrome staining. Immunostaining for ALDH1A1 and mCherry was performed using an anti-ALDH1A1 rabbit polyclonal antibody (dilution factor 1:500; GTX123973, GeneTex, Irvine, CA, USA) and anti-mCherry rabbit polyclonal antibody (dilution factor 1:400; ab167453, Abcam, Cambridge, UK), respectively, as the primary antibodies and a peroxidase-conjugated anti-rabbit goat IgG polyclonal antibody (ready to use; #424144, Nichirei, Tokyo, Japan) as the secondary antibody.

### Statistical analyses

All experiments were performed 2 or 3 times and the representative data are shown as median ± interquartile range except for mRNA data, which is shown as mean ± SEM to ensure the visibility of the graph. The Kruskal–Wallis test for median values was used to assess the statistical significance between groups. Correlation coefficients for parameters were calculated using the Spearman’s rank correlation coefficient analysis. Kaplan–Meier analysis and log-rank test were used for survival analysis. A *P* value < 0.05 was considered significant. All statistical analyses were performed using JMP Pro 14 (SAS Institute Inc., Cary, NC, USA).

## Results

### ***Detection of ALDH***^***br***^*** in mouse lung***

**Fig. 1:**
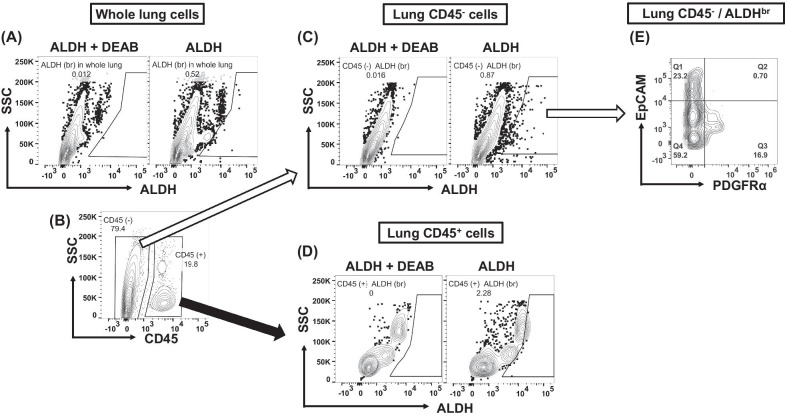
Detection of cell population with high ALDH activity in the mouse lung. Lung cells obtained from 6-to-8-week-old wild-type C57BL/6 mice were analyzed using flow cytometry. Data were analyzed using the FACS Diva (BD Biosciences) and FlowJo (version 10.7.1, BD Biosciences) software. **A** ALDH^br^ gating determined by comparing samples stained with ALDEFLUOR alone and with ALDEFLUOR and diethylaminobenzaldehyde (DEAB), a specific inhibitor of ALDH. **B**–**D** ALDH^br^ cells observed in the whole lung, in the nonhematopoietic/lung-resident CD45^−^ fraction, and in the hematopoietic CD45^+^ fraction. **E** CD45^−^/ALDH^br^ cells divided further into EpCAM^+^ and PDGFRα^+^ fractions

Following the determination of the appropriate ALDH^br^ gating using ALDEFLUOR staining with the DEAB ALDH inhibitor, we observed a rare ALDH^br^ population in the whole lung of mice (Fig. 1A). When we divided the whole lung cells into CD45^+^ hematopoietic cells and CD45^−^ nonhematopoietic cells (Fig. 1B), we noted that both fractions contained ALDH^br^ (Fig. 1C, D). To assess lung resident ALDH^br^, we focused on the nonhematopoietic CD45^−^/ALDH^br^ fraction. Analysis of these nonhematopoietic cells, that is, the lung resident CD45^−^/ALDH^br^ fraction, revealed that this fraction was further divided into mesenchymal (platelet-derived growth factor receptor α positive, PDGFRα^+^) and epithelial (epithelial cell adhesion molecule positive, EpCAM^+^) phenotypes (Fig. 1E).

### ***Characteristics of lung CD45***^***−***^***/ALDH***^***br***^

**Fig. 2:**
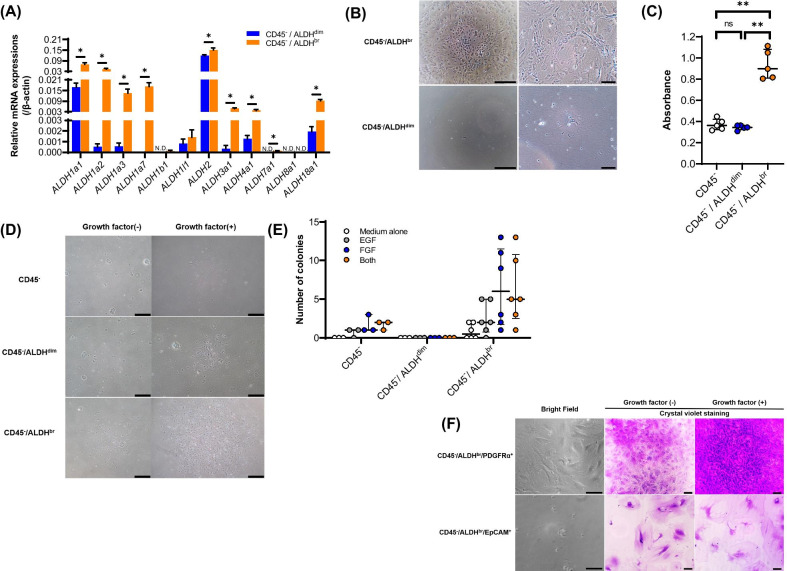
**A** Real-time quantitative PCR analysis of the mRNA expression levels of ALDH isoforms in sorted CD45^−^/ALDH^br^ (*n* = 3) and CD45^−^/ALDH^dim^ cells (cell population with low ALDH activity, *n* = 3). Values are expressed relative to the expression of the endogenous control β-actin mRNA. Data are shown as mean ± SEM. *P < 0.05, N.D., not detectable. **B** Representative image of cultured cells (left, low-power field, scale bar, 400 µm, right, high-power field, scale bar, 100 µm). **C** The proliferative capacity of cells using a CCK-8 kit (*n* = 5/group), as shown in (**B**). **P < 0.01. **D** Representative image of colonies derived from the colony-forming assay using MethoCult supplemented with or without growth factors. Scale bar, 400 µm. **E** The number of colonies derived from the colony-forming assay in (**D**). **F** Representative image of sorted cell populations cultured with or without growth factors. The bright field and cells stained with crystal violet are shown. Scale bar, 70 µm. **G** Sorted CD45^−^/ALDH^br^/PDGFRα^+^ cells induced for differentiation into adipocytes and stained with Oil red O. Scale bar, 70 µm

**Fig. 3:**
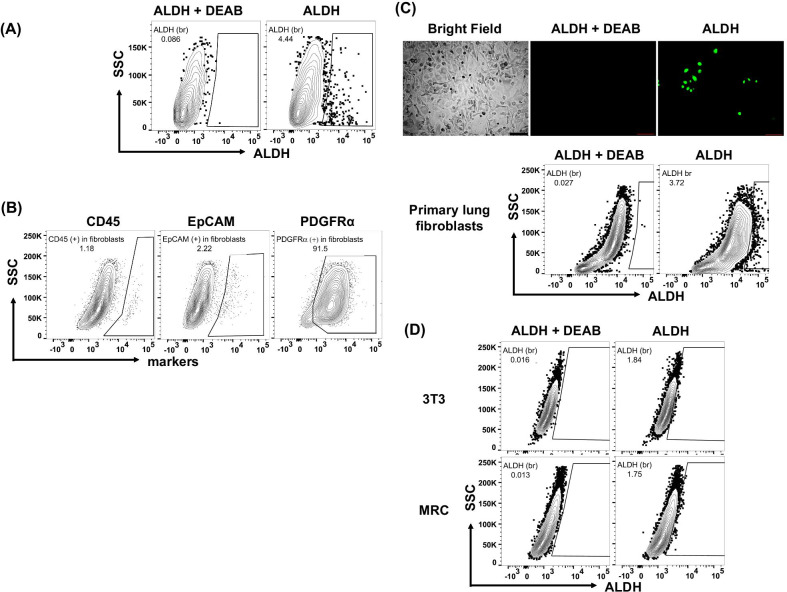
ALDH activity in cultured cells. **A** ALDH activity in cultured lung CD45^−^/ALDH^br^. Sorted CD45^−^/ALDH^br^ cells were cultured and reexamined for ALDH activity. **B** Surface markers on primary cultured lung fibroblasts. **C** ALDH activity in primary cultured lung fibroblasts. (Upper) Representative image of primary cultured lung fibroblasts stained with either ALDEFLUOR alone or ALDEFLUOR and DEAB. (Lower) Flow cytometry to determine ALDH activity in primary cultured lung fibroblasts. **C** ALDH activity in 3T3 and MRC fibroblast cell lines

To determine the characteristics of lung CD45^−^/ALDH^br^, we collected CD45^−^/ALDH^br^ (*n* = 3) and CD45^−^/ALDH^dim^ (*n* = 3) cells using FACS. As shown in Additional file 3, pre-depletion of unnecessary cell populations prior to FACS resulted in the enrichment of the CD45^−^/ALDH^br^ fraction. To confirm if sorted CD45^−^/ALDH^br^ cells truly expressed high levels of ALDH mRNA and to determine the isoforms of ALDH that were mainly expressed in CD45^−^/ALDH^br^ cells, we performed real-time quantitative PCR. Our results showed that the levels of mRNA expression of *ALDH1a*, *ALDH2*, *ALDH3a1*, *ALDH4a1*, *ALDH7a1*, and *ALDH18a* were significantly higher in the CD45^−^/ALDH^br^ than in the CD45^−^/ALDH^dim^ cells (Fig. 2A, *P* = 0.049 for *ALDH1a1*, *P* = 0.049 for *ALDH1a2*, *P* = 0.049 for *ALDH1a3*, *P* = 0.037 for *ALDH1a7*, *P* = 0.049 for *ALDH2*, *P* = 0.046 for *ALDH3a1*, *P* = 0.049 for *ALDH4a1*, *P* = 0.037 for *ALDH7a1*, and *P* = 0.049 for *ALDH18a*). We further observed that when both cell populations were cultured, the CD45^−^/ALDH^br^ population showed higher proliferative ability than the CD45^−^/ALDH^dim^ population (Fig. 2B, C, *P* = 0.009 between CD45^−^ and CD45^−^/ALDH^br^ and *P* = 0.009 between CD45^−^/ALDH^dim^ and CD45^−^/ALDH^br^). To examine whether CD45^−^/ALDH^br^ cells maintained a high ALDH activity in culture, sorted CD45^−^/ALDH^br^ cells were further cultured, harvested, and reexamined for ALDH activity. As shown in Fig. 3A, most proliferated cells were ALDH^dim^, with ALDH^br^ cells accounting for approximately 5% of the total proliferative cells.

Next, we examined the colony-forming ability of CD45^−^/ALDH^br^ cells using a colony-forming assay. We found that CD45^−^/ALDH^br^ cells formed larger (Fig. 2D) and higher number (Fig. 2E) of colonies than the CD45^−^/ALDH^dim^ cells. Although a similar pattern of colony formation was observed for CD45^−^, the size and the number of colonies were relatively small, suggesting that the colony-forming ability of the CD45^−^ population depended to a large extent on the CD45^−^/ALDH^br^ cells. As the CD45^−^/ALDH^br^ population seemed to be a heterogeneous cell population and ALDH^br^ is associated with stemness in various tissues, we evaluated the expression of surface antigens associated with the mesenchymal cells, fibroblasts, and the stem cells in the CD45^−^/ALDH^br^ population. As shown in Additional file 4, not all CD45^−^/ALDH^br^ cells expressed the representative markers of bone marrow-derived MSCs (CD44, CD73, CD90, and CD105). It was notable that the stage-specific embryonic antigen-4 (SSEA4) stem cell marker was solely expressed in CD45^−^/ALDH^br^ cells in the mouse lung.

When we divided the CD45^−^/ALDH^br^ population into CD45^−^/ALDH^br^/PDGFRα^+^ and CD45^−^/ALDH^br^/EpCAM^+^ population, and investigated their characteristics, we observed that the CD45^−^/ALDH^br^/PDGFRα^+^ population exhibited a fibroblast-like spindle shape, whereas the CD45^−^/ALDH^br^/EpCAM^+^ population exhibited a flat and round shape (Fig. 2F). We also found that growth factors led to an increase in the number of CD45^−^/ALDH^br^/PDGFRα^+^ cells but not that of CD45^−^/ALDH^br^/EpCAM^+ cells^, suggesting that CD45^−^/ALDH^br^/PDGFRα^+^ fraction contributed to the high proliferative potential of the CD45^−^/ALDH^br^ population (Fig. 2F).

Next, we examined the expression of ALDH in primary cultured lung fibroblasts (Fig. 3A). These primary cultured lung fibroblasts obtained from BLM-untreated wild-type C57BL/6 mice were shown to frequently express PDGFRα, but not CD45 or EpCAM, suggesting that these cells were truly fibroblasts (Fig. 3B). As shown in Fig. 3C, both fluorescent microscopy and flow cytometry revealed that the percentage of ALDH^br^ cells in primary cultured lung fibroblasts was approximately 5%. Similarly, we noted that the percentage of ALDH^br^ cells in fibroblast cell lines was also less than 5% (Fig. 3D).

### ***Kinetics of CD45***^***−***^***/ALDH***^***br***^*** in BLM-induced pulmonary fibrosis***

**Fig. 4:**
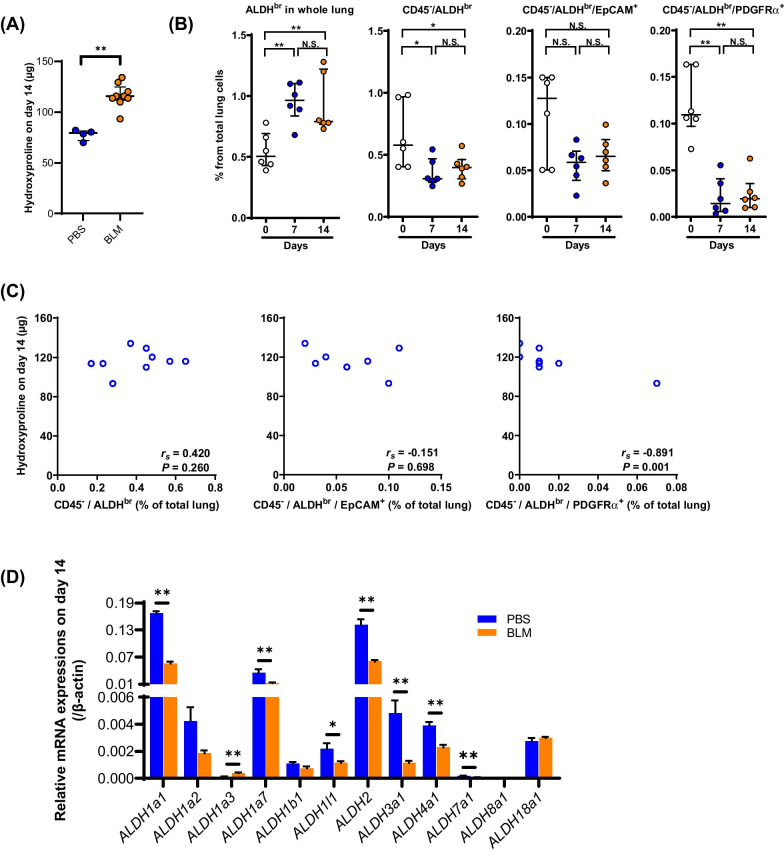
Decreased CD45^−^/ALDH^br^ during pulmonary fibrosis. **A** Lung hydroxyproline content on day 14 in the PBS (*n* = 4) and the BLM (*n* = 9) groups. **P < 0.01. **B** Percentages of ALDH^br^, CD45^−^/ALDH^br^, CD45^−^/ALDH^br^/EpCAM^+^, and CD45^−^/ALDH^br^/PDGFRα^+^ cells in total lung cells during BLM-induced pulmonary fibrosis (*n* = 6/group). *P < 0.05, **P < 0.01. ns, not significant. **C** Correlations between lung hydroxyproline content on day 14 and percentages of ALDH^br^, CD45^−^/ALDH^br^, CD45^−^/ALDH^br^/EpCAM^+^, and CD45^−^/ALDH^br^/PDGFRα^+^ cells in total lung cells. **D** Real-time quantitative PCR analysis of the mRNA expression levels of ALDH isoforms in the lungs obtained from the PBS (*n* = 4) and BLM (*n* = 9) groups on day 14. Values are expressed relative to the expression of the endogenous control β-actin mRNA. Data are shown as mean ± SEM. *P < 0.05, **P < 0.01

To investigate the kinetics of CD45^−^/ALDH^br^ in fibrotic lungs, we used endotracheal administration of BLM (2 mg/kg body weight) to generate a mouse model of pulmonary fibrosis. We found that the levels of hydroxyproline were significantly elevated in the BLM group (*n* = 9) 14 days after BLM administration (Fig. 4A, *P* = 0.005) compared with the PBS group (*n* = 4). On days 7 and 14, the percentage of total ALDH^br^ cells in the lung was significantly elevated compared with that on day 0 (Fig. 4B), whereas the percentage of CD45^−^/ALDH^br^ cells and CD45^−^/ALDH^br^/PDGFRα^+^ cells, but not CD45^−^/ALDH^br^/EpCAM^+^ cells, were significantly decreased (Fig. 4B). Among the ALDH^br^ populations in the lung obtained on day 14, the percentage of CD45^−^/ALDH^br^/PDGFRα^+^ cells, but not that of CD45^−^/ALDH^br^ cells or CD45^−^/ALDH^br^/EpCAM^+^ cells, inversely correlated with the levels of hydroxyproline (Fig. 4C).

Real-time quantitative PCR analysis revealed that the mRNA expression of *ALDH1a1*, *ALDH1a7*, *ALDH1l1*, *ALDH2*, *ALDH3a1*, *ALDH4a1*, and *ALDH7a1* was significantly lower in the fibrotic lung obtained on day 14 (Fig. 4D, *P* = 0.006 for *ALDH1a1*, *P* = 0.006 for *ALDH1a7*, *P* = 0.014 for *ALDH1l1*, *P* = 0.006 for *ALDH2*, *P* = 0.006 for *ALDH3a1*, *P* = 0.006 for *ALDH4a1*, and *P* = 0.009 for *ALDH7a1*). Consistent with the reduced number of CD45^−^/ALDH^br^ cells and the reduced expression of *ALDH1a1* mRNA in the fibrotic lung, the expression of ALDH1a1 was reduced throughout the alveolar epithelia, especially in the areas of fibrosis, as demonstrated using immunostaining (Additional file 5).

The decrease in number of cells observed in CD45^−^/ALDH^br^ cells during BLM treatment was a feature observed in lung SP cells as well [10]. Therefore, we investigated the possibility of an overlap between ALDH^br^ and lung SP cells. After Hoechst staining, ALDH staining was performed, followed by flow cytometry, which revealed that CD45^−^/ALDH^br^ population is completely different from CD45^−^ lung SP cells (Additional file 6).

### ***Effect of CD45***^***−***^***/ALDH***^***br***^*** cell therapy on BLM-induced pulmonary fibrosis***

**Fig. 5:**
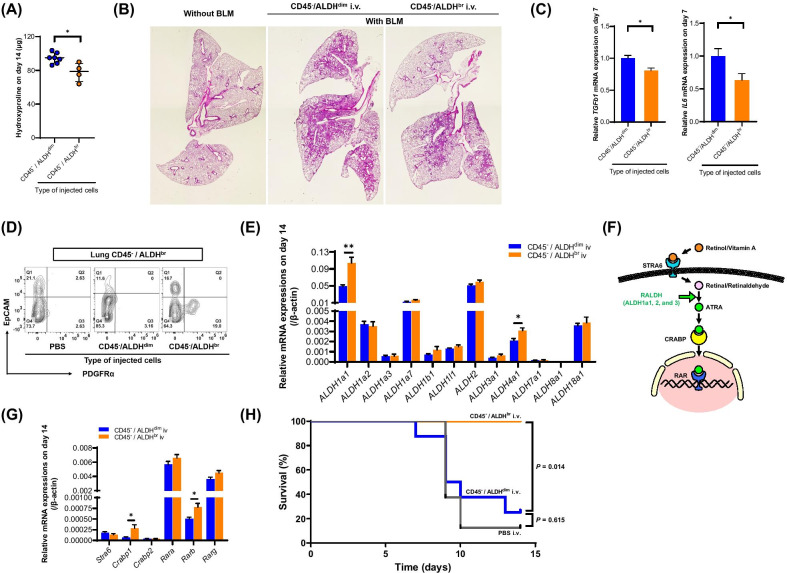
CD45^−^/ALDH^br^ cell therapy ameliorates BLM-induced pulmonary fibrosis CD45^−^/ALDH^br^ cell therapy ameliorates BLM-induced pulmonary fibrosis. **A** Lung hydroxyproline content on day 14 in BLM-treated mice transferred with CD45^−^/ALDH^dim^ (*n* = 7) and CD45^−^/ALDH^br^ (*n* = 4) cells on day 2. *P < 0.05. **B** HE-staining of lung tissue sections obtained from PBS-treated and BLM-treated mice transferred with CD45^−^/ALDH^dim^ and CD45^−^/ALDH^br^ cells. **C** Real-time quantitative PCR analysis of the mRNA expression levels of TGF-β1 and IL-6 in lung tissues on day 7 obtained from BLM-treated mice transferred with CD45^−^/ALDH^dim^ (*n* = 9–10) and CD45^−^/ALDH^br^ (*n* = 7–8) cells. Values are expressed relative to the expression of the endogenous control β-actin mRNA and normalized to the mean value of the CD45^−^/ALDH^dim^ i.v. group set as 1. *P < 0.05. **D** Representative images of flow cytometry of lung CD45^−^/ALDH^br^ cells in BLM-treated mice transferred with PBS alone, CD45^−^/ALDH^dim^ cells, and CD45^−^/ALDH^br^ cells on day 14. **E** Real-time quantitative PCR analysis of the mRNA expression levels of ALDH isoforms in the lungs obtained from BLM-treated mice transferred with CD45^−^/ALDH^dim^ (*n* = 7) and CD45^−^/ALDH^br^ (*n* = 4) cells on day 14. Values are expressed relative to the expression of endogenous control β-actin mRNA. Data are shown as mean ± SEM. *P < 0.05. **F** Schematic summary of the retinol-metabolizing pathway. Circulating retinol/vitamin A is taken up into a cell via the cell membrane receptor (stimulated by retinoic acid 6, STRA6). It is converted into retinal/retinaldehyde within the cytoplasm, which is further converted into all-trans-retinoic acid (ATRA) by retinal dehydrogenase/retinaldehyde dehydrogenase (RALDH, ALDH1a family). Subsequently, it is transported into the nucleus by the cellular retinoic acid-binding protein (CRABP), where ATRA binds to the intranuclear retinoic acid receptor (RAR) and promotes gene transcription. **G** Real-time quantitative PCR analysis of the mRNA expression levels of retinol-metabolizing pathway-related genes in the lung tissues obtained from BLM-treated mice transferred with CD45^−^/ALDH^dim^ (*n* = 7) and CD45^−^/ALDH^br^ (*n* = 4) cells on day 14. Values are expressed relative to the expression of the endogenous control β-actin mRNA. Data are shown as mean ± SEM. *P < 0.05. (H) Survival rate in high-dose BLM-treated mice transferred with PBS alone (*n* = 8), CD45^−^/ALDH^dim^ (*n* = 8) cells, and CD45^−^/ALDH^br^ (*n* = 5) cells

In the preceding experiments, we presumed that CD45^−^/ALDH^br^ cells were depleted during pulmonary fibrosis; therefore, we assessed the possible usage of CD45^−^/ALDH^br^ cells in cell therapy for BLM-induced pulmonary fibrosis. Our results showed that both the levels of hydroxyproline (Fig. 5A, *P* = 0.023) and the degree of tissue fibrosis (Fig. 5B for HE staining, Additional file 7 for Masson’s trichrome staining) in the lung obtained on day 14 were significantly lower in the CD45^−^/ALDH^br^ i.v. group (*n* = 4) than in the CD45^−^/ALDH^dim^ i.v. group (*n* = 7). In the CD45^−^/ALDH^br^ i.v. group (*n* = 7–8), the mRNA expression of interleukin 6 (*IL6*) and transforming growth factor β1 (*TGFb1*) genes in lung tissues obtained on day 7 was significantly suppressed compared with the CD45^−^/ALDH^dim^ i.v. group (*n* = 9–10) (Fig. 5C, *P* = 0.042 for *IL6*, and *P* = 0.013 for *TGFb1*). Interestingly, we noted that the percentage of CD45^−^/ALDHbr/PDGFRα^+^ cells, which was lowered, was recovered in the CD45^−^/ALDH^br^ i.v. group (*n* = 4) in the lung obtained on day 14 (Fig. 5D). Furthermore, the expression levels of *ALDH1a1* and *ALDH4a1* mRNAs, which were significantly reduced after treatment with BLM (Fig. 4D), were also recovered in the CD45^−^/ALDH^br^ i.v. group (*n* = 4) on day 14 (Fig. 5E, *P* = 0.008 for *ALDH1a1*, and *P* = 0.038 for *ALDH4a1*).

Among the ALDH family of enzymes, the ALDH1a family (ALDH1a1, ALDH1a2, and ALDH1a3), also known as retinal dehydrogenases or retinaldehyde dehydrogenases (RALDH), convert retinal/retinaldehyde to all-trans retinoic acid (ATRA) (Fig. 5F) [22]. To investigate the effect of the transferred CD45^−^/ALDH^br^ cells on the retinol-metabolizing pathway in BLM-induced pulmonary fibrosis, we examined the retinol-metabolizing pathway-related mRNA expression in BLM-treated lung tissue. As shown in Fig. 5G, the mRNA expression levels of cellular retinoic acid-binding protein 1 (*Crabp1*) and retinoic acid receptor beta (*Rarb*) were significantly increased in the CD45^−^/ALDH^br^ i.v. group (*n* = 4) compared with the CD45^−^/ALDH^dim^ i.v. group (*n* = 7) (*P* = 0.019 for *Crabp1*, and *P* = 0.038 for *Rarb*).

In addition, we assessed the effect of CD45^−^/ALDH^br^ cell therapy on survival using BLM-induced pulmonary fibrosis with a higher dose of BLM (5 mg/kg body weight). We observed that the higher dose of BLM led to approximately 80% mortality on day 14 in both the CD45^−^/ALDH^dim^ (*n* = 8) and the PBS i.v. groups (*n* = 8), whereas, surprisingly, no death was observed in mice that received CD45^−^/ALDH^br^ cell therapy (*n* = 5) (Fig. 5H).

### ***Detection of transferred donor CD45***^***−***^***/ALDH***^***br***^*** in the recipient lung***

**Fig. 6:**
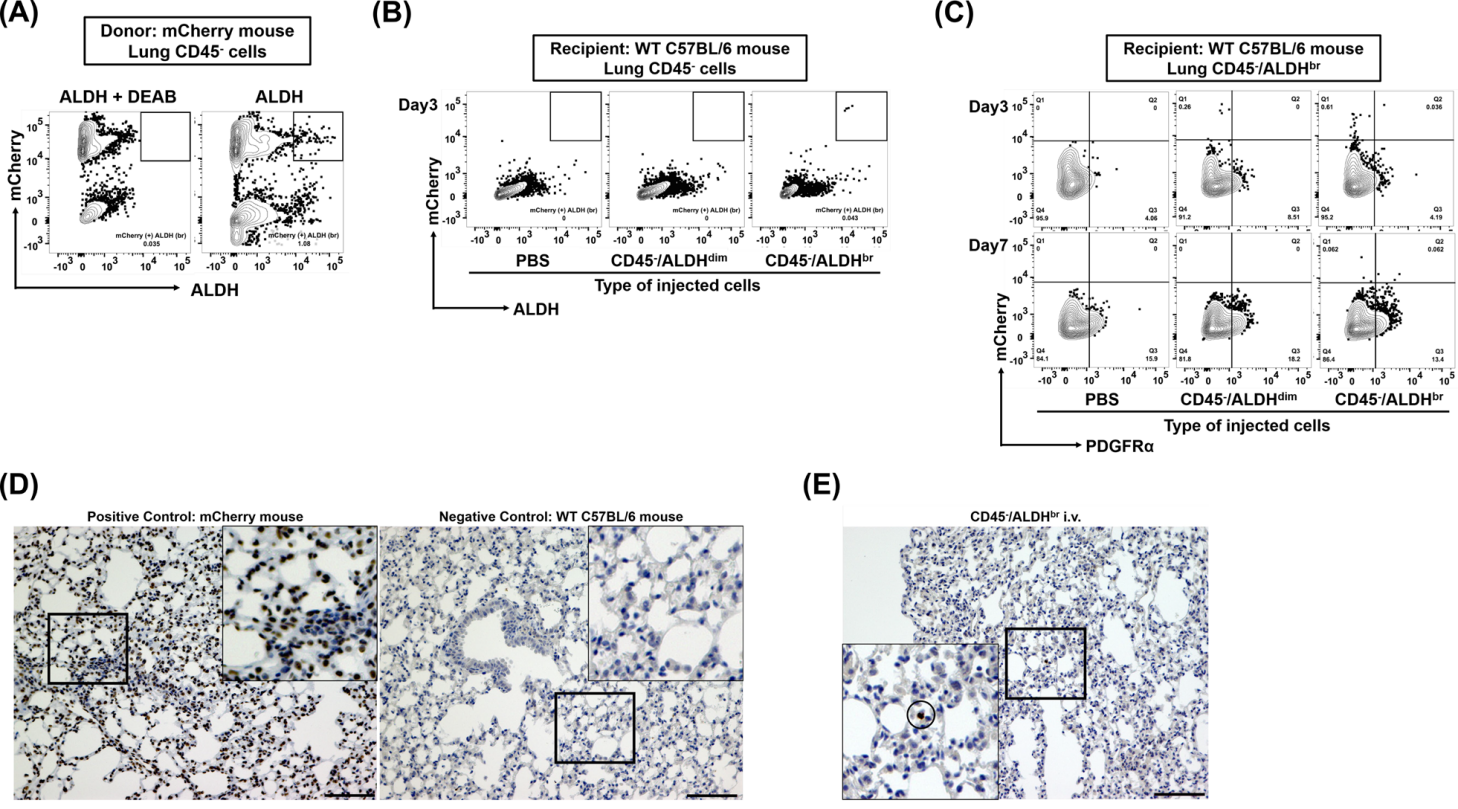
Detection of transferred donor CD45^−^/ALDH^br^ cells in the recipient lung. **A** Representative images of flow cytometry of lung CD45^−^ cells in donor mCherry-expressing mice. **B** Flow cytometry of lung CD45^−^ cells in recipient BLM-treated C57BL/6 mice transferred with PBS alone, donor mCherry^+^/CD45^−^/ALDH^dim^ cells, and donor mCherry^+^/CD45^−^/ALDH^br^ cells. Recipient lungs were harvested on the day following the cell transfer (day 3, 3 days after BLM-treatment). **C** Flow cytometry of lung CD45^−^/ALDH^br^ cells in recipient BLM-treated C57BL/6 mice transferred with PBS alone, donor mCherry^+^/CD45^−^/ALDH^dim^ cells, and donor mCherry^+^/CD45^−^/ALDH^br^ cells. Recipient lungs were harvested the next day and 5 days after cell transfer (days 3 and 7). **D** Histological analysis of mCherry immunostaining in lungs obtained from mCherry-expressing mice (positive control) and wild-type C57BL/6 mice (negative control). **E** Representative images of mCherry immunostaining in lungs obtained from recipient BLM-treated wild-type C57BL/6 mice transferred with mCherry^+^/CD45^−^/ALDH^br^ cells

To distinguish and trace the injected donor CD45^−^/ALDH^br^ cells in the lungs of recipient mice, mCherry knock-in mice were used as donors. After mCherry heterozygosity was confirmed using tail PCR (Additional file 8), donor CD45^−^/ALDH^br^ or CD45^−^/ALDH^dim^ cells were sorted from these mCherry-expressing mice using FACS (Fig. 6A) and transferred into wild-type C57BL/6 recipients pretreated with BLM. We observed that flow cytometry could detect donor mCherry-positive CD45^−^/ALDH^br^ (Fig. 6B) and CD45^−^/ALDH^br^/PDGFRα^+^ (Fig. 6C) cells in the recipient lungs more frequently in the CD45^−^/ALDH^br^ i.v. group than in the CD45^−^/ALDH^dim^ i.v. group. Appropriate mCherry immunostaining conditions were determined using appropriate positive and negative controls (Fig. 6D), and we noted that mCherry-positive CD45^−^/ALDH^br^ and CD45^−^/ADLH^br^/PDGFRα^+^ cells were also found histologically in the recipient lung-transferred CD45^−^/ALDH^br^ (Fig. 6E).

### Effects of aging on ALDH activity

**Fig. 7:**
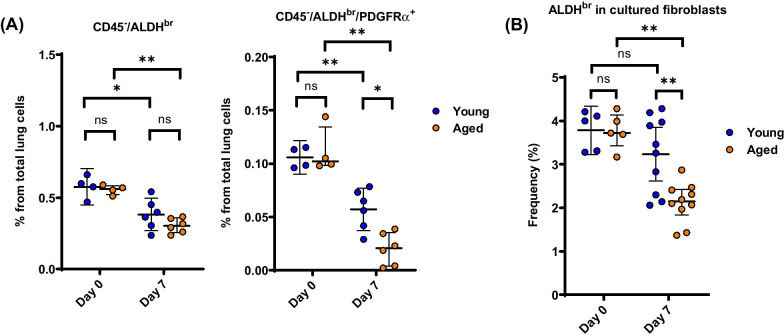
Effect of aging on ALDH activity. **A** Percentages of CD45^−^/ALDH^br^ and CD45^−^/ALDH^br^/PDGFRα^+^ cells in total lung cells during BLM-induced pulmonary fibrosis in young and aged mice (*n* = 4–6). *P < 0.05, **P < 0.01. ns, not significant. **B** ALDH activity in primary cultured lung fibroblasts obtained from young and aged mice (*n* = 5–10) before and 7 days after treatment with BLM. **P < 0.01. ns, not significant

Finally, we examined the role of aging on the CD45^−^/ALDH^br^ population. As shown in Fig. 7A, the percentages of whole CD45^−^/ALDH^br^ cell population and that of its CD45^−^/ALDH^br^/PDGFRα^+^ subgroup in the lung were not significantly different between aged and young mice that were not treated with BLM (on day 0). On the contrary, the percentage of CD45^−^/ALDH^br^/PDGFRα^+^ cells, but not CD45^−^/ALDH^br^ cells, in the lung obtained 7 days after treatment with BLM was significantly decreased in aged mice (*n* = 4–6) compared with that in young mice (*n* = 4–6). In a similar fashion, the percentage of ALDH^br^ cells in cultured PDGFRα-predominant (as shown in Fig. 3B) primary lung fibroblasts obtained from the lung 7 days after treatment with BLM was significantly decreased in aged mice (*n* = 5–10) compared with young mice (*n* = 5–10) (Fig. 7B).

## Discussion

The present study identified and characterized the nonhematopoietic/lung resident ALDH^br^ cell populations in the mouse lung. The lung CD45^−^/ALDH^br^ population and, the CD45^−^/ALDH^br^/PDGFRα^+^ subpopulation are cell populations with high proliferative capacity. These population significantly reduced in pulmonary fibrosis. The high levels of expression of ALDH observed in CD45^−^/ALDH^br^ cells was mainly attributed to the ALDH1a subfamily, also known as RALDH, which was significantly reduced in BLM-treated lungs. When used as a tool for cell therapy, transferred CD45^−^/ALDH^br^ cells reached the site of lung injury and ameliorated BLM-induced pulmonary fibrosis. Thus, this study demonstrated CD45^−^/ALDH^br^ cells as a novel lung-resident stem cell population and suggested their potential therapeutic use in pulmonary fibrosis.

Although ALDH^br^ cells with stem cell properties have been detected in various normal tissues, including the bone marrow [14, 23], umbilical cord blood [24, 25], mammary glands [26, 27], heart [28], and adipose tissue [29], little is known about lung-resident ALDH^br^ cells. A study showed that isolated murine airway basal and submucosal gland duct ALDH^br^ cells exhibited stem cell properties in normal/healthy lungs [30]. No previous study has investigated the significance of lung-resident ALDH^br^ cells in respiratory diseases, such as pulmonary fibrosis. In the current study, lung-resident CD45^−^/ALDH^br^ were rare and heterogeneous population with epithelial and mesenchymal lineages. The percentages of ALDH^br^ in both primary cultured lung fibroblasts and fibroblast cell lines were low at approximately 5%, and hence, we assumed that ALDH^br^ cells lost their activity during differentiation and proliferation, consistent with the findings of a previous report [24]. Similar to the CD45^−^/ALDH^br^/PDGFRα^+^ population in the current study, CD45^−^ lung SP cells have been reported to express mesenchymal markers and exhibit MSC properties [8], and have been shown to be decreased in BLM-induced pulmonary fibrosis [10]. However, in our study, we found that CD45^−^/ALDH^br^ is a novel population that is completely different from lung SP cells (CD45^−^/CD31^−^/Hoechst^dim^). Therefore, it is reasonable that the expression of the surface markers of MSCs found in lung CD45^−^/ALDH^br^ cells differed from that in the SP cells. Instead, SSEA4, a marker for mesenchymal progenitors [31], was demonstrated to be solely expressed on CD45^−^/ALDH^br^ cells in the mouse lung. These results suggest that the CD45^−^/ALDH^br^ population might contain mesenchymal progenitors and CD45^−^/ALDH^br^/PDGFR^+^ cells maintained the ability to differentiate into the mesenchymal lineage.

During BLM-induced pulmonary fibrosis, we observed a downregulation in the expression of a broad spectrum of ALDH mRNAs in lung tissues. We also found that transferred CD45^−^/ALDH^br^ cells ameliorated BLM-induced pulmonary fibrosis by suppressing IL-6 and TGF-β. As an evidence, intravenously administered CD45^−^/ALDH^br^ cells were shown to reach the site of lung injury using mCherry-expressing mice as donors. Additionally, these lung-protective effects of transferred CD45^−^/ALDH^br^ were accompanied by a recovery in the levels of ALDH, which had been decreased during fibrosis, suggesting that ALDH was involved in the mechanism of pulmonary fibrosis. Although little is known about the association of ALDH isoforms with lung diseases, ALDH1a1 and ALDH3a1 have been reported to be expressed in the human airway epithelium [32]. Jang and coworkers reported that the expression of ALDH3a1 was markedly increased in human airway epithelial cells exposed to cigarette smoke extract and that ALDH3a1 exerted protective action against smoking-induced airway epithelial damage [33]. In the current study, the expression of both ALDH1a1 and ALDH4a1 were upregulated in CD45^−^/ALDH^br^ cells and downregulated in the fibrotic lung after BLM administration, paralleling the reduction in the number of CD45^−^/ALDH^br^ cells. Likewise, intravenous administration of CD45^−^/ALDH^br^ cells was shown to significantly recover the expression of ALDH1a1 and ALDH4a1 in the fibrotic lung. Therefore, we speculated that mesenchymal ALDH1a1 and ALDH4a1 might protect against BLM-induced pulmonary fibrosis. Indeed among ALDH family members, RALDHs (ALDH1a1, ALDH1a2, and ALDH1a3) catalyze the conversion of retinol to ATRA [22], supporting the self-renewal and cell differentiation of stem cells [34]. Several lines of evidence have suggested that ATRA exerted protective action against radiation pneumonitis and BLM-induced lung injury in mice through anti-inflammatory effects by activating protein kinase C δ (PKC-δ), inhibiting mitogen-activated protein kinase P38 α (p38MAPK) and nuclear factor kappa-light-chain-enhancer of activated B-cells (NF-kB), and suppressing the production of IL-6 and TGF-β [35–38]. In the current study, we observed the upregulation of retinol-metabolizing pathway molecules, recovery of the expression of RALDH, and suppressed expression of IL-6 and TGF-β in BLM-induced pulmonary fibrosis treated with CD45^−^/ALDH^br^ cell therapy. On the other hand, the significance of ALDH4a1 in lung injury is currently unknown and further investigation is required.

In the fibrotic lung, after BLM administration, we observed a reduction in the number of cells in the CD45^−^/ALDH^br^ population, especially of its CD45^−^/ALDH^br^/PDGFRα^+^ subpopulation. This reduction was more remarkably observed in aged mice than in young mice. These results suggested that aging led to a decrease in the number of ALDH^br^ cells in the lungs, especially in the lung PDGFRα^+^ fibroblasts. As fibrotic lung diseases, especially idiopathic pulmonary fibrosis (IPF), commonly occur in the elderly [39] and stem cell senescence is one of the suggested causes of IPF [40], it is speculated that the decreased number of ALDH^br^ cells in the lungs might accelerate fibrotic lung diseases in the elderly.

The limitation of this study is the difficulty in collecting sufficient number of cells. Because of the infrequency of existence of ALDH^br^ cells, many donor mice lungs were necessary to acquire a sufficient number of ALDH^br^ cells, signifying the challenge in applying the methods and the results of the present study to human lung diseases. If the collected cells could be proliferated while maintaining ALDH activity, the burden on donor could be minimized. To apply the current results to human translational studies in the future, development of less invasive methods for collecting ALDH^br^ cells is required.

## Conclusions

**Fig. 8:**
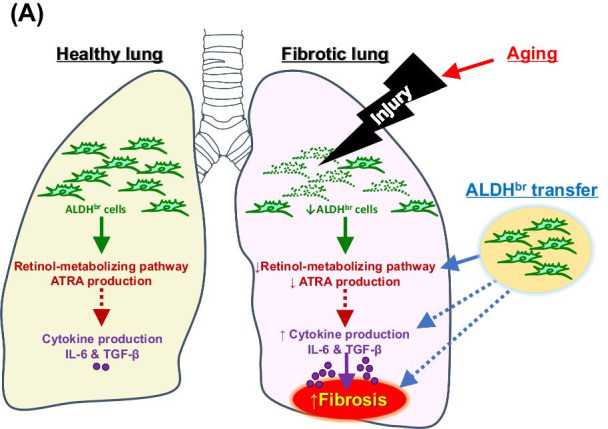
Summary of the study. Lung injury (e.g., bleomycin-induced lung injury) triggers reduction of ALDH^br^ cells in the lung, resulting in a suppressed retinol-metabolizing pathway, elevated concentrations of profibrotic cytokines (e.g., IL-6 and TGFβ1), and exacerbation of pulmonary fibrosis. Aging accelerates the injury-induced reduction in ALDH^br^ cells. ALDH^br^ cell therapy restores the impaired antifibrotic effects of ALDH^br^ cells. Solid and dotted arrows indicate promotion and inhibition, respectively

Our results strongly suggest that the lung expression of ALDH and lung-resident CD45^−^/ALDH^br^ are involved in pulmonary fibrosis. (Figure 8 summarizes the findings of the current study.) When administered intravenously, CD45^−^/ALDH^br^ ameliorated BLM-induced pulmonary fibrosis, signifying the possibility for CD45^−^/ALDH^br^ cells to find application as novel and useful cell therapy tools in pulmonary fibrosis treatment.

## Supplementary Information

**Additional file 1.** Antibodies used in flow cytometric experiments.

**Additional file 2.** Primers used in this study.

**Additional file 3.** Effect of magnetic-activated cell sorting for the enrichment of CD45^−^/ALDH^br^ cells. Representative image of lung CD45^−^/ALDH^br^ cells before and after magnetic-activated cell sorting using the Tissue Stem Cell Pre-Enrichment Kit. ALDH^br^ gating was determined by comparing samples stained with ALDEFLUOR alone and with ALDEFLUOR and diethylaminobenzaldehyde (DEAB), an ALDH inhibitor.

**Additional file 4.** Expression of cell surface proteins in CD45^−^/ALDH^dim^ cells and CD45^−^/ALDH^br^ cells. Sorted CD45^−^/ALDH^dim^ cells and CD45^−^/ALDH^br^ cells were examined for cell surface markers associated with mesenchymal stem cells (MSCs), fibroblasts, and stem cells.

**Additional file 5.** ALDH1a1 immunostaining in lung tissue. Representative images of ALDH1a1 immunostaining in PBS- or BLM-treated lung tissue sections on day 14.

**Additional file 6.** Double staining of ALDH^br^ cells and side population cells in lung tissue. (A) Representative image of lung CD45^−^/CD31^−^/ALDH^br^ live cells. (B) Representative image of lung CD45^−^/CD31^−^/Hoechst^dim^ (side population, SP) live cells. (C) The gating of SP cells was confirmed using verapamil, which is an inhibitor for Hoechst staining. (D) SP cells in lung CD45^−^/CD31^−^/ALDH^br^ live cells. (E) ALDH^br^ cells in SP cells.

**Additional file 7.** CD45^−^/ALDH^br^ cell therapy ameliorates BLM-induced pulmonary fibrosis. Masson’s trichrome staining of lung tissue sections obtained on day 14 from BLM-treated mice transferred with CD45^−^/ALDH^dim^ and CD45^−^/ALDH^br^ cells.

**Additional file 8.** Discrimination of mCherry-heterozygotic mouse. DNA extracted from the tails of mice was amplified by PCR using primers shown in Table S2. The expression of the mCherry-heterozygotic band was evaluated using agarose gel electrophoresis.

## Data Availability

The data that support the findings of this study are available from the corresponding author upon reasonable request.

## Abbreviations

ALDH: Aldehyde dehydrogenase
ALDH^br^: Cell populations with high ALDH activity
ALDH^dim^: Cell population with low ALDH activity
ATRA: All-trans retinoic acid
BLM: Bleomycin
CRABP: Cellular retinoic acid-binding protein
EpCAM: Epithelial cell adhesion molecule
FACS: Fluorescence activated cell sorting
HE: Hematoxylin–eosin
IL: Interleukin
IPF: Idiopathic pulmonary fibrosis
MSCs: Mesenchymal stem cells
NF-kB: Nuclear factor kappa-light-chain-enhancer of activated B-cells
PDGFR: Platelet-derived growth factor receptor
PKC-δ: Protein kinase C δ
p38MAPK: Mitogen-activated protein kinase P38 α
RALDH: Retinal dehydrogenase/retinaldehyde dehydrogenase
RAR: Retinoic acid receptor
SP: Side population
SSEA: Stage-specific embryonic antigen
TGF: Transforming growth factor

## References

[1] Wecht S, Rojas M (2016). Mesenchymal stem cells in the treatment of chronic lung disease. Respirology.

[2] Iyer SS, Rojas M (2008). Anti-inflammatory effects of mesenchymal stem cells: novel concept for future therapies. Expert Opin Biol Ther.

[3] Rojas M, Iyer SS, Torres-Gonzalez E, Neujahr DC, Kwon M, Brigham KL (2010). Effect of bone marrow-derived mesenchymal stem cells on endotoxin-induced oxidation of plasma cysteine and glutathione in mice. Stem Cells Int..

[4] Kyurkchiev D (2014). Secretion of immunoregulatory cytokines by mesenchymal stem cells. World J Stem Cells.

[5] Rojas M, Xu J, Woods CR, Mora AL, Spears W, Roman J (2005). Bone marrow-derived mesenchymal stem cells in repair of the injured lung. Am J Respir Cell Mol Biol.

[6] Hegab AE, Kubo H, Fujino N, Suzuki T, He M, Kato H (2010). Isolation and characterization of murine multipotent lung stem cells. Stem Cells Dev.

[7] Majka SM, Beutz MA, Hagen M, Izzo AA, Voelkel N, Helm KM (2005). Identification of novel resident pulmonary stem cells: form and function of the lung side population. Stem Cells.

[8] Martin J, Helm K, Ruegg P, Varella-Garcia M, Burnham E, Majka S (2008). Adult lung side population cells have mesenchymal stem cell potential. Cytotherapy.

[9] Summer R, Fitzsimmons K, Dwyer D, Murphy J, Fine A (2007). Isolation of an adult mouse lung mesenchymal progenitor cell population. Am J Respir Cell Mol Biol.

[10] Jun D, Garat C, West J, Thorn N, Chow K, Cleaver T (2011). The pathology of bleomycin-induced fibrosis is associated with loss of resident lung mesenchymal stem cells that regulate effector T-cell proliferation. Stem Cells.

[11] Muzio G, Maggiora M, Paiuzzi E, Oraldi M, Canuto RA (2012). Aldehyde dehydrogenases and cell proliferation. Free Radic Biol Med.

[12] Balber AE (2011). Concise review: aldehyde dehydrogenase bright stem and progenitor cell populations from normal tissues: characteristics, activities, and emerging uses in regenerative medicine. Stem Cells.

[13] Capoccia BJ, Robson DL, Levac KD, Maxwell DJ, Hohm SA, Neelamkavil MJ (2009). Revascularization of ischemic limbs after transplantation of human bone marrow cells with high aldehyde dehydrogenase activity. Blood.

[14] Gentry T, Foster S, Winstead L, Deibert E, Fiordalisi M, Balber A (2007). Simultaneous isolation of human BM hematopoietic, endothelial and mesenchymal progenitor cells by flow sorting based on aldehyde dehydrogenase activity: implications for cell therapy. Cytotherapy.

[15] Sondergaard CS, Hess DA, Maxwell DJ, Weinheimer C, Rosová I, Creer MH (2010). Human cord blood progenitors with high aldehyde dehydrogenase activity improve vascular density in a model of acute myocardial infarction. J Transl Med.

[16] Nakashima T, Liu T, Hu B, Wu Z, Ullenbruch M, Omori K (2019). Role of B7H3/IL-33 signaling in pulmonary fibrosis-induced profibrogenic alterations in bone marrow. Am J Respir Crit Care Med.

[17] Ueda J, Maehara K, Mashiko D, Ichinose T, Yao T, Hori M (2014). Heterochromatin dynamics during the differentiation process revealed by the DNA methylation reporter mouse, methylRO. Stem Cell Rep.

[18] Alison MR, Guppy NJ, Lim SML, Nicholson LJ (2010). Finding cancer stem cells: are aldehyde dehydrogenases fit for purpose?. J Pathol.

[19] Summer R, Kotton DN, Sun X, Ma B, Fitzsimmons K, Fine A (2003). Side population cells and Bcrp1 expression in lung. Am J Physiol Lung Cell Mol Physiol.

[20] Nakashima T, Liu T, Yu H, Ding L, Ullenbruch M, Hu B (2013). Lung bone marrow-derived hematopoietic progenitor cells enhance pulmonary fibrosis. Am J Respir Crit Care Med.

[21] Sisson TH, Hanson KE, Subbotina N, Patwardhan A, Hattori N, Simon RH (2002). Inducible lung-specific urokinase expression reduces fibrosis and mortality after lung injury in mice. Am J Physiol Lung Cell Mol Physiol.

[22] Ma I, Allan AL (2011). The role of human aldehyde dehydrogenase in normal and cancer stem cells. Stem Cell Rev..

[23] Armstrong L, Stojkovic M, Dimmick I, Ahmad S, Stojkovic P, Hole N (2004). Phenotypic characterization of murine primitive hematopoietic progenitor cells isolated on basis of aldehyde dehydrogenase activity. Stem Cells.

[24] Seneviratne AK, Bell GI, Sherman SE, Cooper TT, Putman DM, Hess DA (2016). Expanded hematopoietic progenitor cells reselected for high aldehyde dehydrogenase activity demonstrate islet regenerative functions. Stem Cells.

[25] Nagano M, Yamashita T, Hamada H, Ohneda K, Kimura KI, Nakagawa T (2007). Identification of functional endothelial progenitor cells suitable for the treatment of ischemic tissue using human umbilical cord blood. Blood.

[26] Ginestier C, Hur MH, Charafe-Jauffret E, Monville F, Dutcher J, Brown M (2007). ALDH1 is a marker of normal and malignant human mammary stem cells and a predictor of poor clinical outcome. Cell Stem Cell.

[27] Douville J, Beaulieu R, Balicki D (2009). ALDH1 as a functional marker of cancer stem and progenitor cells. Stem Cells Dev.

[28] Roehrich ME, Spicher A, Milano G, Vassalli G (2013). Characterization of cardiac-resident progenitor cells expressing high aldehyde dehydrogenase activity. Biomed Res Int.

[29] Itoh H (2017). Aldehyde dehydrogenase activity helps identify a subpopulation of murine adipose-derived stem cells with enhanced adipogenic and osteogenic differentiation potential. World J Stem Cells.

[30] Hegab AE, Ha VL, Darmawan DO, Gilbert JL, Ooi AT, Attiga YS (2012). Isolation and in vitro characterization of basal and submucosal gland duct stem/progenitor cells from human proximal airways. Stem Cells Transl Med.

[31] Xia H, Bodempudi V, Benyumov A, Hergert P, Tank D, Herrera J (2014). Identification of a cell-of-origin for fibroblasts comprising the fibrotic reticulum in idiopathic pulmonary fibrosis. Am J Pathol.

[32] Patel M, Lu L, Zander DS, Sreerama L, Coco D, Moreb JS (2008). ALDH1A1 and ALDH3A1 expression in lung cancers: correlation with histologic type and potential precursors. Lung Cancer Irel.

[33] Jang JH, Bruse S, Liu Y, Duffy V, Zhang C, Oyamada N (2014). Aldehyde dehydrogenase 3A1 protects airway epithelial cells from cigarette smoke-induced DNA damage and cytotoxicity. Free Radic Biol Med.

[34] Khillan JS (2014). Vitamin A/retinol and maintenance of pluripotency of stem cells. Nutrients.

[35] Tabata C, Kadokawa Y, Tabata R, Takahashi M, Okoshi K, Sakai Y (2006). All-trans-retinoic acid prevents radiation- or bleomycin-induced pulmonary fibrosis. Am J Respir Crit Care Med.

[36] Dong Z, Tai W, Yang Y, Zhang T, Li Y, Chai Y (2012). The role of all-trans retinoic acid in bleomycin-induced pulmonary fibrosis in mice. Exp Lung Res.

[37] Song X, Liu W, Xie S, Wang M, Cao G, Mao C (2013). All-transretinoic acid ameliorates bleomycin-induced lung fibrosis by downregulating the TGF-β1/Smad3 signaling pathway in rats. Lab Investig..

[38] Leem AY, Shin MH, Douglas IS, Song JH, Chung KS, Kim EY (2017). All-trans retinoic acid attenuates bleomycin-induced pulmonary fibrosis via downregulating EphA2–EphrinA1 signaling. Biochem Biophys Res Commun.

[39] Wijsenbeek M, Cottin V (2020). Spectrum of fibrotic lung diseases. N Engl J Med.

[40] Aldera JK, Barkauskas CE, Limjunyawong N, Stanley SE, Kembou F, Tuder RM (2015). Telomere dysfunction causes alveolar stem cell failure. Proc Natl Acad Sci USA.

