# Supplementary material for: Antifibrotic effect of lung-resident progenitor cells with high aldehyde dehydrogenase activity
Source: Stem Cell Res Ther. 2021 Aug 23;12:471. doi: 10.1186/s13287-021-02549-6 (PMC8381511; doi:10.1186/s13287-021-02549-6)
Supplement: Supplementary file 1 — Additional file 1. Antibodies used in flow cytometric experiments. [file 13287_2021_2549_MOESM1_ESM.docx]

**Additional file 1: Antibodies used in flow cytometric experiments**

| **Antibody** | **Clone** | **Conjugation** |
| --- | --- | --- |
| CD16/32 | 93 | None |
| CD45 | 30-F11 | Pacific blue |
| CD45 | 30-F11 | PerCP/Cy5.5 |
| EpCAM/CD326 | G8.8 | PE-Cy7 |
| PDGFRα/CD140a | APA5 | APC |
| PDGFRβ/CD140b | APB5 | APC |
| CD31 | MEC13.3 | PerCP/Cy5.5 |
| CD44 | IM7 | PE |
| CD73 | TY/11.8 | PE |
| CD90.2 | 30-H12 | PE |
| CD105 | MJ7/18 | APC |
| SSEA3 | MC-631 | PE |
| SSEA4 | MC-813-70 | PE |
