# Supplementary material for: Antifibrotic effect of lung-resident progenitor cells with high aldehyde dehydrogenase activity
Source: Stem Cell Res Ther. 2021 Aug 23;12:471. doi: 10.1186/s13287-021-02549-6 (PMC8381511; doi:10.1186/s13287-021-02549-6)
Supplement: Supplementary file 2 — Additional file 2. Primers used in this study. [file 13287_2021_2549_MOESM2_ESM.docx]

**Additional file 2: Primers used in this study**

Primers for quantitative PCR

| **Gene Name** | **Assay ID** |
| --- | --- |
| *ALDH1a1* | Mm00657317_m1 |
| *ALDH1a2* | Mm00501306_m1 |
| *ALDH1a3* | Mm00474049_m1 |
| *ALDH1a7* | Mm00496380_m1 |
| *ALDH1b1* | Mm00728303_s1 |
| *ALDH1l1* | Mm03048957_m1 |
| *ALDH2* | Mm00477463_m1 |
| *ALDH3a1* | Mm00839312_m1 |
| *ALDH4a1* | Mm00615268_m1 |
| *ALDH7a1* | Mm00519645_m1 |
| *ALDH8a1* | Mm00724562_m1 |
| *ALDH18a1* | Mm00444767_m1 |
| *Tgfb1* | Mm03024053_m1 |
| *Il6* | Mm00446190_m1 |
| *Stra6* | Mm00486457_m1 |
| *Crabp1* | Mm00442775_g1 |
| *Crabp2* | Mm00801693_g1 |
| *Rara* | Mm01296312_m1 |
| *Rarb* | Mm01319677_m1 |
| *Rarg* | Mm00441091_m1 |

Primers for qualitative PCR to distinguish mCherry heterozygotic mice

| **Name** | **Sequence** | **Length** |
| --- | --- | --- |
| ROSA26-NotI-Fw | GAGCGGCCGCCCACCCTCCCCTTCCTCTGG | 30 |
| ROSA26-NruI-Rev | CCTCGCGACACTGTATTTCATACTGTAGTA | 30 |
| EGFP-Rev | TTACTTGTACAGCTCGTCCATGCCG | 25 |
