# Supplementary material for: Antifibrotic effect of lung-resident progenitor cells with high aldehyde dehydrogenase activity
Source: Stem Cell Res Ther. 2021 Aug 23;12:471. doi: 10.1186/s13287-021-02549-6 (PMC8381511; doi:10.1186/s13287-021-02549-6)
Supplement: Supplementary file 3 — Additional file 3. Effect of magnetic-activated cell sorting for the enrichment of CD45−/ALDHbr cells. Representative image of lung CD45−/ALDHbr cells before and after magnetic-activated cell sorting using the Tissue Stem Cell Pre-Enrichment Kit. ALDHbr gating was determined by comparing samples stained with ALDEFLUOR alone and with ALDEFLUOR and diethylaminobenzaldehyde (DEAB), an ALDH inhibitor. [file 13287_2021_2549_MOESM3_ESM.pptx]

## Slide 1
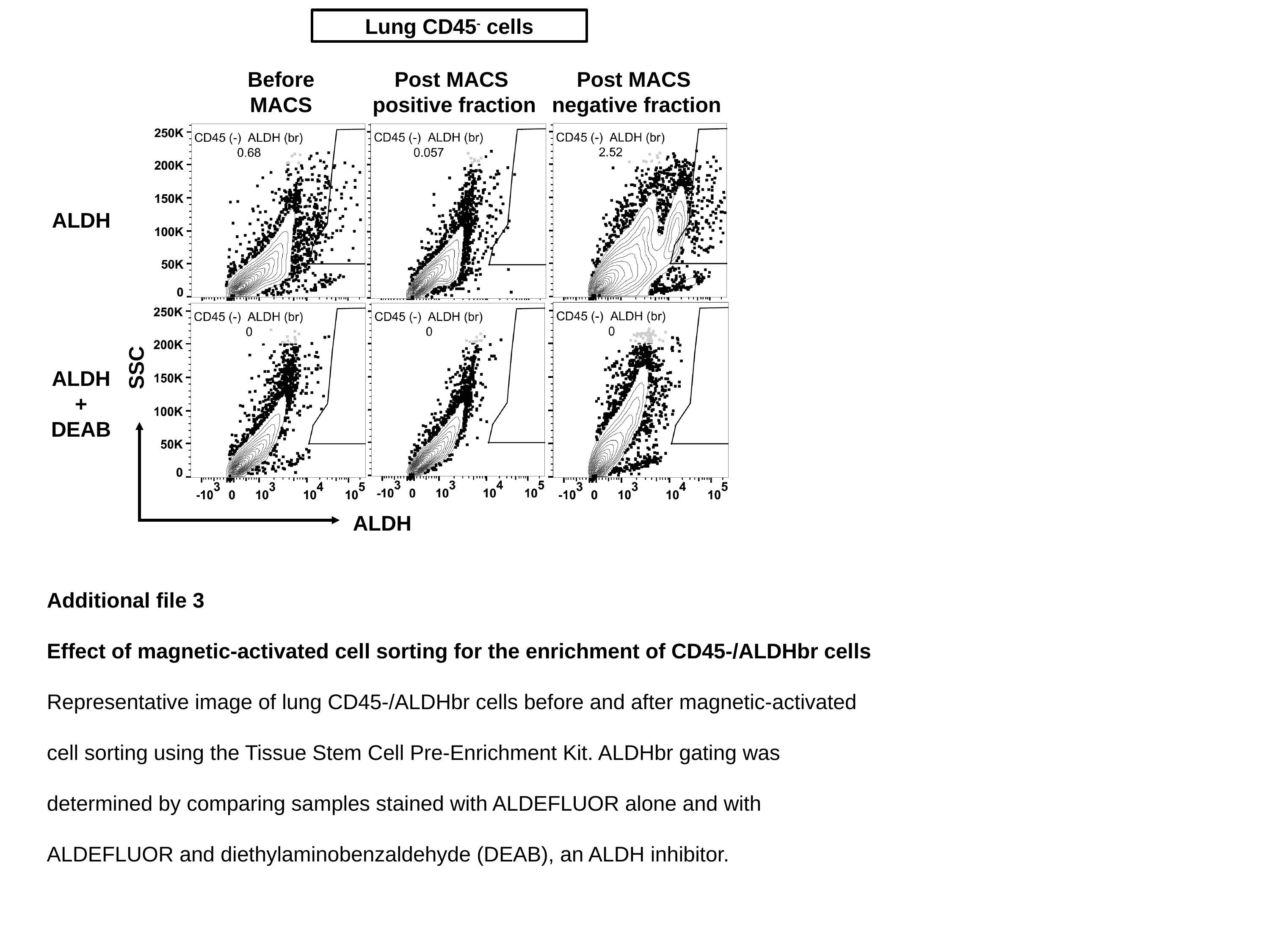

Lung CD45- cells
Before MACS
Post MACS
positive fraction
Post MACS
negative fraction
ALDH
SSC
ALDH
ALDH
+
DEAB
Additional file 3
Effect of magnetic-activated cell sorting for the enrichment of CD45-/ALDHbr cells
Representative image of lung CD45-/ALDHbr cells before and after magnetic-activated cell sorting using the Tissue Stem Cell Pre-Enrichment Kit. ALDHbr gating was determined by comparing samples stained with ALDEFLUOR alone and with ALDEFLUOR and diethylaminobenzaldehyde (DEAB), an ALDH inhibitor.
