# Supplementary material for: Antifibrotic effect of lung-resident progenitor cells with high aldehyde dehydrogenase activity
Source: Stem Cell Res Ther. 2021 Aug 23;12:471. doi: 10.1186/s13287-021-02549-6 (PMC8381511; doi:10.1186/s13287-021-02549-6)
Supplement: Supplementary file 4 — Additional file 4. Expression of cell surface proteins in CD45−/ALDHdim cells and CD45−/ALDHbr cells. Sorted CD45−/ALDHdim cells and CD45−/ALDHbr cells were examined for cell surface markers associated with mesenchymal stem cells (MSCs), fibroblasts, and stem cells. [file 13287_2021_2549_MOESM4_ESM.pptx]

## Slide 1
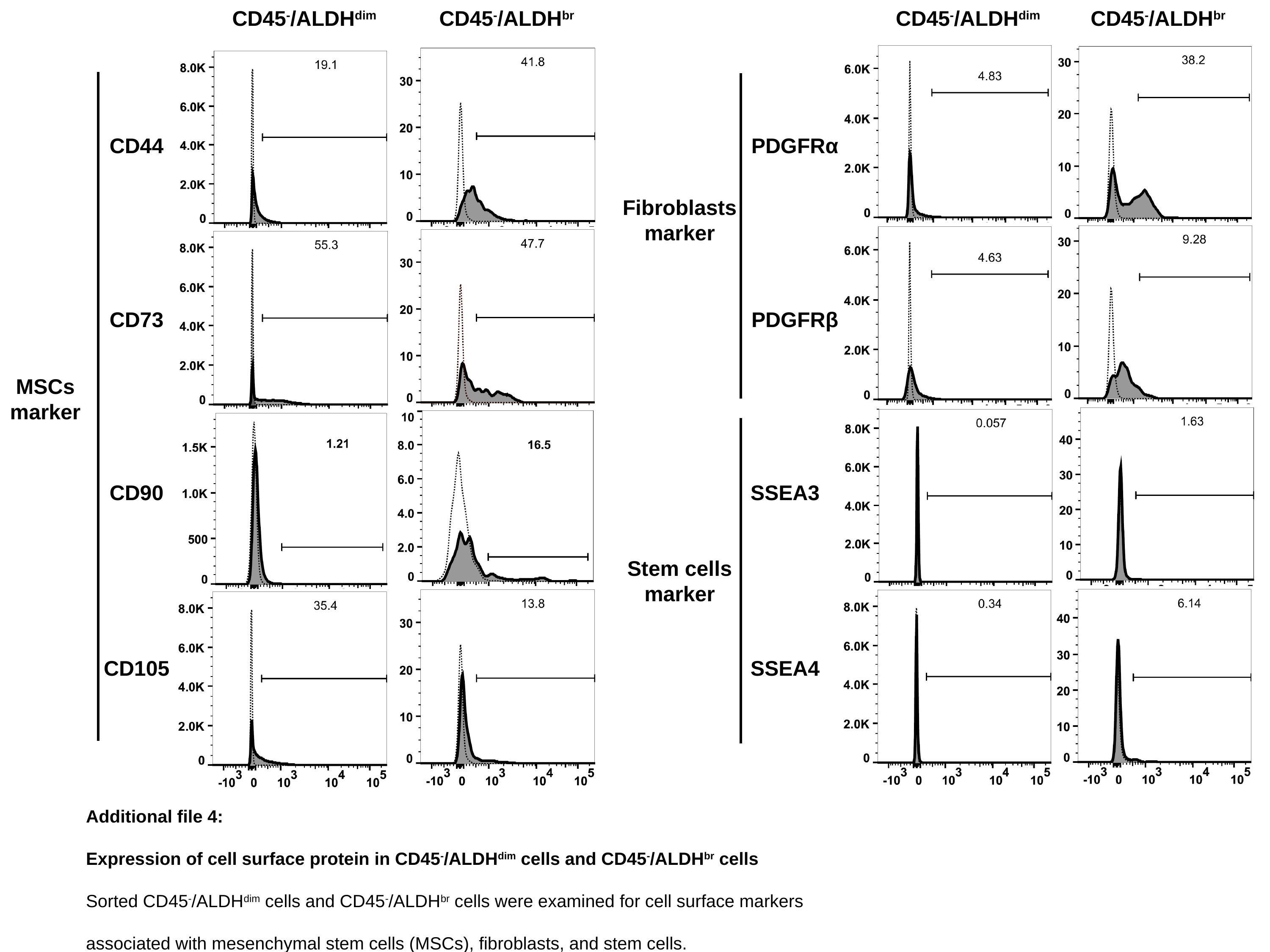

CD45-/ALDHdim
CD45-/ALDHbr
CD45-/ALDHdim
CD45-/ALDHbr
CD44
PDGFRα
Fibroblasts
marker
CD73
PDGFRβ
MSCs
marker
CD90
SSEA3
Stem cells
marker
CD105
SSEA4
Additional file 4:
Expression of cell surface protein in CD45-/ALDHdim cells and CD45-/ALDHbr cells
Sorted CD45-/ALDHdim cells and CD45-/ALDHbr cells were examined for cell surface markers associated with mesenchymal stem cells (MSCs), fibroblasts, and stem cells.
