# Supplementary material for: Antifibrotic effect of lung-resident progenitor cells with high aldehyde dehydrogenase activity
Source: Stem Cell Res Ther. 2021 Aug 23;12:471. doi: 10.1186/s13287-021-02549-6 (PMC8381511; doi:10.1186/s13287-021-02549-6)
Supplement: Supplementary file 5 — Additional file 5. ALDH1a1 immunostaining in lung tissue. Representative images of ALDH1a1 immunostaining in PBS- or BLM-treated lung tissue sections on day 14. [file 13287_2021_2549_MOESM5_ESM.pptx]

## Slide 1
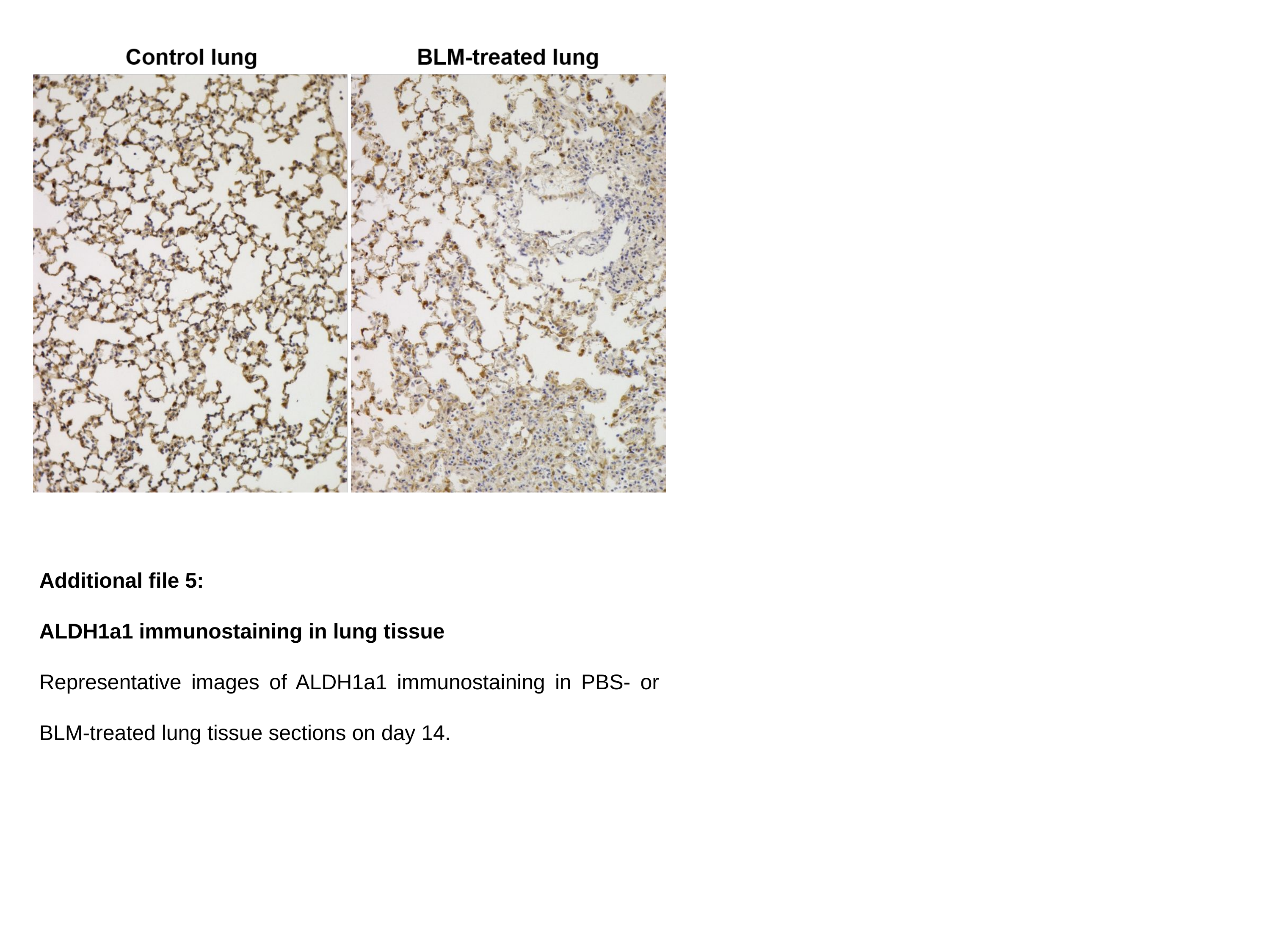

Additional file 5:
ALDH1a1 immunostaining in lung tissue
Representative images of ALDH1a1 immunostaining in PBS- or BLM-treated lung tissue sections on day 14.
