# Supplementary material for: Antifibrotic effect of lung-resident progenitor cells with high aldehyde dehydrogenase activity
Source: Stem Cell Res Ther. 2021 Aug 23;12:471. doi: 10.1186/s13287-021-02549-6 (PMC8381511; doi:10.1186/s13287-021-02549-6)
Supplement: Supplementary file 6 — Additional file 6. Double staining of ALDHbr cells and side population cells in lung tissue. (A) Representative image of lung CD45−/CD31−/ALDHbr live cells. (B) Representative image of lung CD45−/CD31−/Hoechstdim (side population, SP) live cells. (C) The gating of SP cells was confirmed using verapamil, which is an inhibitor for Hoechst staining. (D) SP cells in lung CD45−/CD31−/ALDHbr live cells. (E) ALDHbr cells in SP cells. [file 13287_2021_2549_MOESM6_ESM.pptx]

## Slide 1
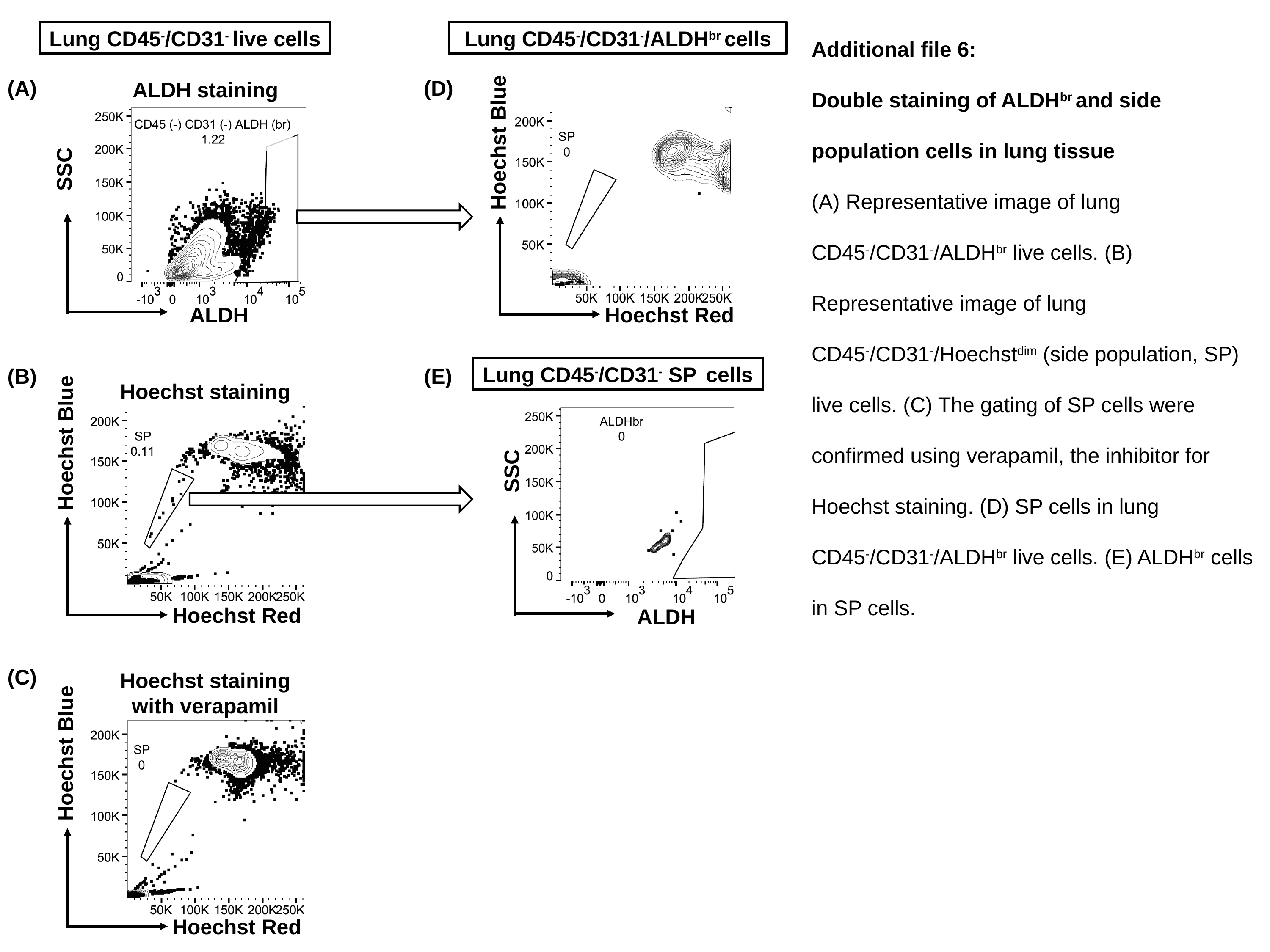

Additional file 6:
Double staining of ALDHbr and side population cells in lung tissue
(A) Representative image of lung CD45-/CD31-/ALDHbr live cells. (B) Representative image of lung CD45-/CD31-/Hoechstdim (side population, SP) live cells. (C) The gating of SP cells were confirmed using verapamil, the inhibitor for Hoechst staining. (D) SP cells in lung CD45-/CD31-/ALDHbr live cells. (E) ALDHbr cells in SP cells.
Lung CD45-/CD31- live cells
Lung CD45-/CD31-/ALDHbr cells
(A)
(D)
ALDH staining
Hoechst Blue
SSC
ALDH
Hoechst Red
Lung CD45-/CD31- SP cells
(B)
(E)
Hoechst Blue
Hoechst staining
SSC
ALDH
Hoechst Red
(C)
Hoechst staining
with verapamil
Hoechst Blue
Hoechst Red
