# Supplementary material for: Antifibrotic effect of lung-resident progenitor cells with high aldehyde dehydrogenase activity
Source: Stem Cell Res Ther. 2021 Aug 23;12:471. doi: 10.1186/s13287-021-02549-6 (PMC8381511; doi:10.1186/s13287-021-02549-6)
Supplement: Supplementary file 7 — Additional file 7. CD45−/ALDHbr cell therapy ameliorates BLM-induced pulmonary fibrosis. Masson’s trichrome staining of lung tissue sections obtained on day 14 from BLM-treated mice transferred with CD45−/ALDHdim and CD45−/ALDHbr cells. [file 13287_2021_2549_MOESM7_ESM.pptx]

## Slide 1
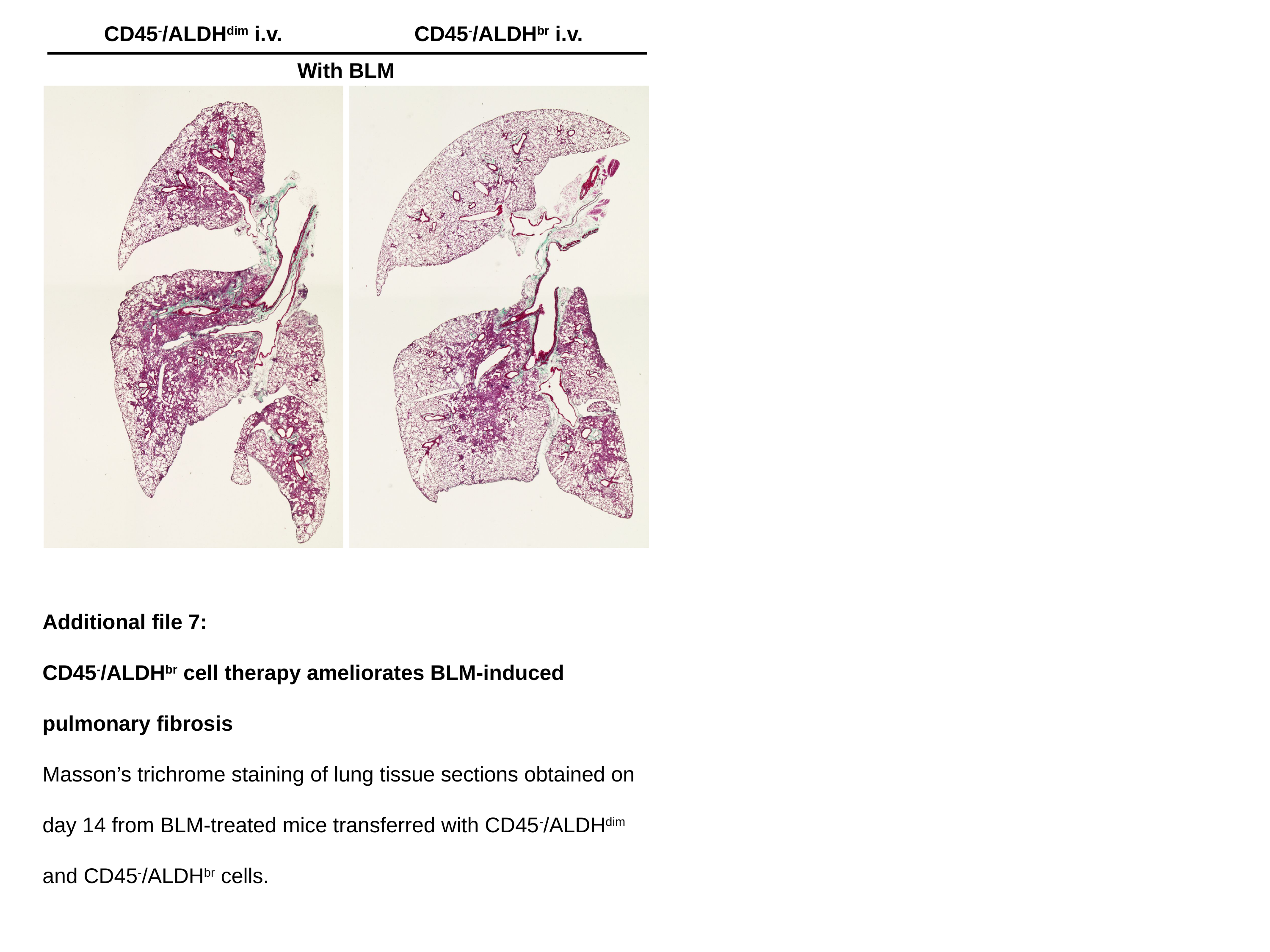

CD45-/ALDHbr i.v.
CD45-/ALDHdim i.v.
With BLM
Additional file 7:
CD45-/ALDHbr cell therapy ameliorates BLM-induced pulmonary fibrosis
Masson’s trichrome staining of lung tissue sections obtained on day 14 from BLM-treated mice transferred with CD45-/ALDHdim and CD45-/ALDHbr cells.
