# Supplementary material for: Antifibrotic effect of lung-resident progenitor cells with high aldehyde dehydrogenase activity
Source: Stem Cell Res Ther. 2021 Aug 23;12:471. doi: 10.1186/s13287-021-02549-6 (PMC8381511; doi:10.1186/s13287-021-02549-6)
Supplement: Supplementary file 8 — Additional file 8. Discrimination of mCherry-heterozygotic mouse. DNA extracted from the tails of mice was amplified by PCR using primers shown in Table S2. The expression of the mCherry-heterozygotic band was evaluated using agarose gel electrophoresis. [file 13287_2021_2549_MOESM8_ESM.pptx]

## Slide 1
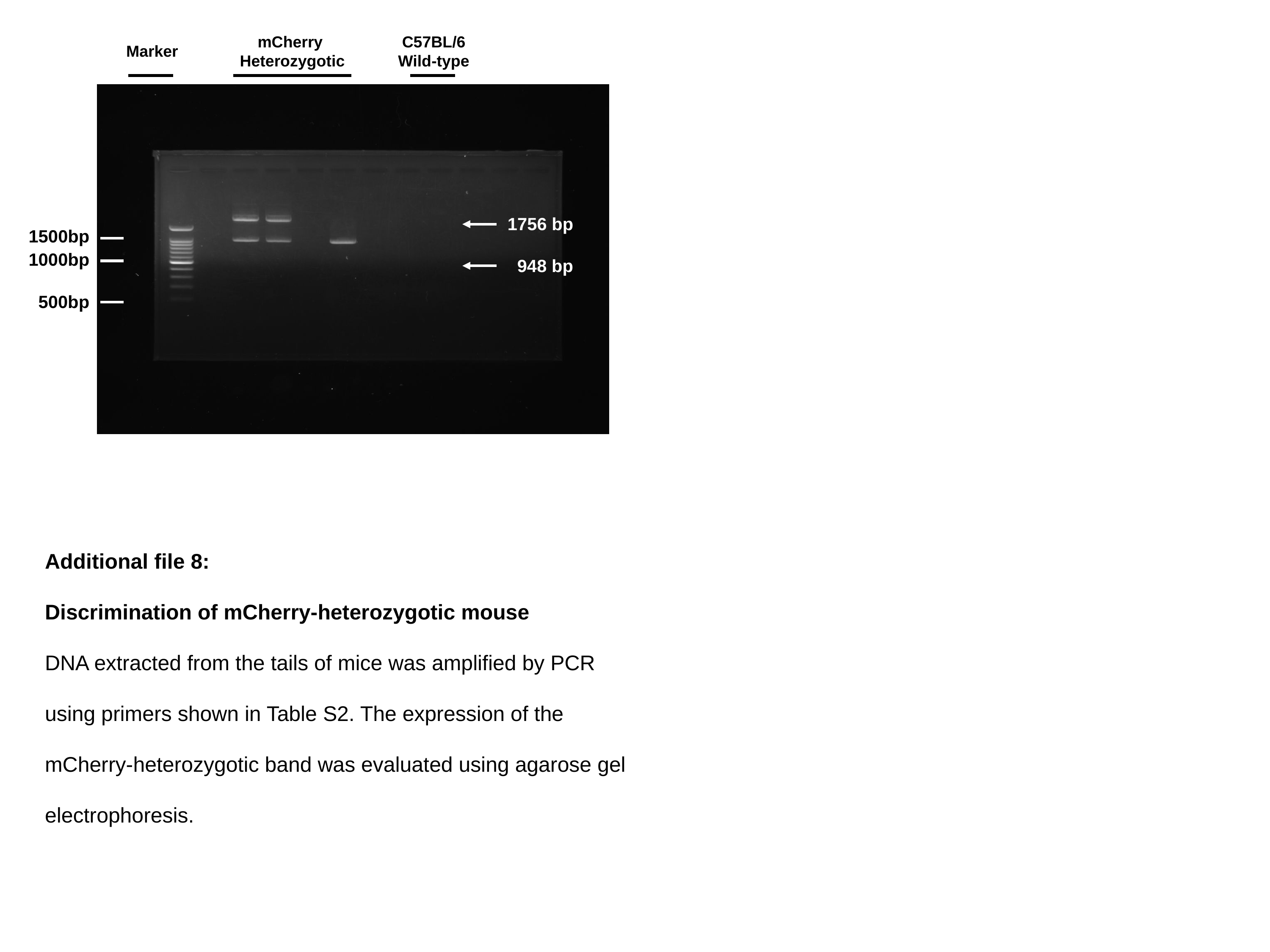

mCherry
Heterozygotic
C57BL/6
Wild-type
Marker
 1756 bp
1500bp
1000bp
 948 bp
500bp
Additional file 8:
Discrimination of mCherry-heterozygotic mouse
DNA extracted from the tails of mice was amplified by PCR using primers shown in Table S2. The expression of the mCherry-heterozygotic band was evaluated using agarose gel electrophoresis.
